# Molecular Mechanisms of Flavonoids against Tumor Gamma-Herpesviruses and Their Correlated Cancers—A Focus on EBV and KSHV Life Cycles and Carcinogenesis

**DOI:** 10.3390/ijms24010247

**Published:** 2022-12-23

**Authors:** Sherif T. S. Hassan, Miroslava Šudomová

**Affiliations:** 1 Department of Applied Ecology, Faculty of Environmental Sciences, Czech University of Life Sciences Prague, Kamýcká 129, 165 00 Prague, Czech Republic; 2 Museum of Literature in Moravia, Klášter 1, 664 61 Rajhrad, Czech Republic

**Keywords:** flavonoids, antiviral activities, anticancer properties, cancer treatment, Epstein–Barr virus (EBV), Kaposi sarcoma-associated herpesvirus (KSHV), tumor viruses, herpesviruses, carcinogenesis, molecular mechanisms

## Abstract

Epstein–Barr virus (EBV) and Kaposi sarcoma-associated herpesvirus (KSHV) are cancer-causing viruses that belong to human gamma-herpesviruses. They are DNA viruses known to establish lifelong infections in humans, with the ability to develop various types of cancer. Drug resistance remains the main barrier to achieving effective therapies for viral infections and cancer. Thus, new medications with dual antiviral and anticancer actions are highly needed. Flavonoids are secondary metabolites biosynthesized by plants with diverse therapeutic effects on human health. In this review, we feature the potential role of flavonoids (flavones, protoflavones, isoflavones, flavanones, flavonols, dihydroflavonols, catechins, chalcones, anthocyanins, and other flavonoid-type compounds) in controlling gamma-herpesvirus-associated cancers by blocking EBV and KSHV infections and inhibiting the formation and growth of the correlated tumors, such as nasopharyngeal carcinoma, Burkitt’s lymphoma, gastric cancer, extranodal NK/T-cell lymphoma, squamous cell carcinoma, Kaposi sarcoma, and primary effusion lymphoma. The underlying mechanisms via targeting EBV and KSHV life cycles and carcinogenesis are highlighted. Moreover, the effective concentrations or doses are emphasized.

## 1. Introduction

Epstein–Barr virus (EBV or human herpesvirus 4) and Kaposi sarcoma-associated herpesvirus (KSHV or human herpesvirus 8) are DNA tumor herpesviruses that belong to the *Gammaherpesvirinae* subfamily. Both viruses have lifelong effects on humans, forming lytic and latent infections, which undergo periodic reactivation. Inducing various types of cancer is the most significant problem reported with both viruses [1,2,3]. Moreover, both viruses can cause neurological complications by affecting the nervous system [4,5]. The human herpesvirus life cycle involves various steps, including attachment to the host cells, entry, lytic replication, latency, and reactivation [6,7]. Briefly, the virus begins the infection by adhering to the host cell via specific binding receptors and enters the cells using multiple viral entry glycoproteins. After the primary infection, the virus induces lytic replication (a multi-step process) and controls the genetic materials of the host cell and its innate immune system [8,9,10]. Subsequently, the virus moves to the latent site, forming latency, and persists in an inactive state. Recurrent infection occurs when the virus is reactivated from a latent state, and this step depends on the function of the infected host’s immune system. The human herpesvirus often reactivates after immunosuppression triggered by diverse physiological and environmental factors that negatively impact the immune system [11,12,13,14,15]. Infections with human gamma-herpesviruses are mostly asymptomatic, and symptoms appear once the immune system is impaired, mainly in immunocompromised patients [16]. Notably, the activation of the lytic cycle of EBV and KSHV leads to the expression of viral genes, proteins, and antigens that could promote tumorigenesis [17]. More information about EBV, KSHV, and their linked cancers is described in later sections.

So far, no drugs have been approved to treat or prevent EBV and KSHV infections effectively; however, acyclovir and its derivatives are currently prescribed to inhibit the replication of both viruses and reduce the severity and frequency of symptoms [18,19]. Moreover, the formed cancers triggered by both viruses are treated by different approaches that involve anticancer medications [20]. Nevertheless, the development of drug resistance makes the therapy for both viruses and their associated malignancies ineffective [3,21]. Therefore, the search for new sources, such as plants, that offer medicines with the ability to overcome this obstacle is highly required [22,23,24].

Consequently, this review aims to shed light on the potential of flavonoids as effective drugs against EBV and KSHV infections and their linked tumors, with a special emphasis on molecular mechanisms that affect the viral life cycle and carcinogenesis. Effective concentrations or doses are included as key factors for evaluating drug effectiveness. We performed a comprehensive literature search covering the period from 2012 to September 2022 (ten years update) using several online databases and search engines, such as Web of Science Core Collection, Scopus, PubMed, ScienceDirect, Embase, SciFinder, and Google Scholar with keywords that define anti-EBV and anti-KSHV properties of flavonoids and their protective potential against the correlated cancers. We have also included a few studies published before 2012 to provide a deeper comparison, critique, and assessment.

## 2. Flavonoids: An Overview, Anti-Herpesvirus, and Anticancer Properties

**Figure 1:**
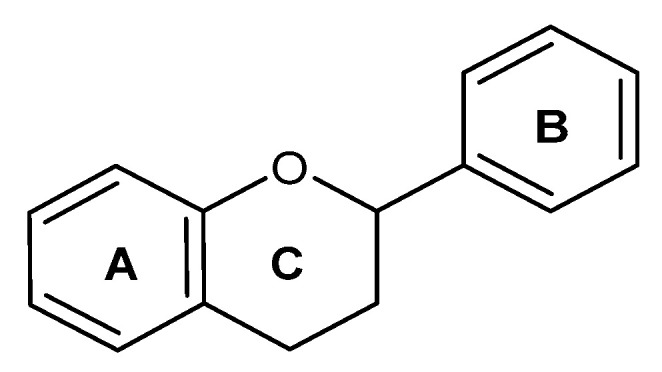
The basic structure of flavonoids.

Flavonoids are a group of bioactive compounds biosynthesized by plants, including edible plants. They belong to the class of polyphenols with a basic skeleton that contains 15-carbon atoms (C6–C3–C6) (Figure 1) [25,26]. Functionally, flavonoids participate in the development process of plants and protect them from diverse biotic and abiotic stresses, including, but not limited to, pathogens and UV radiation damage [27,28]. Chemically, they are classified into several subclasses, such as flavones, protoflavones, isoflavones, flavanones, flavonols, flavanols (including catechins), chalcones, and anthocyanins/anthocyanidins [29,30]. Some factors were found to affect the main structure of the flavonoid molecule, including the degree and pattern of alkalization, hydroxylation, prenylation, or glycosylation reactions [31,32]. The amount of flavonoids in plants varies, and might be influenced by different agricultural and environmental factors, including abiotic stresses. Moreover, the extraction methods can impact the amount yielded [33,34].

Flavonoids are known for their promising health benefits in preventing or curing different health conditions [35]. For instance, flavonoids were found to provide a valuable contribution to herpesvirus research, where these compounds showed an excellent capacity to block the infections of various types of herpesvirus by diverse mechanisms that target multiple stages of the viral life cycle. Inhibition of viral DNA replication was observed to be the main target of flavonoids [36,37,38]. In laboratory, animal, and clinical investigations, flavonoids were also observed to possess notable anticancer properties against different types of cancer cells, with the ability to stop them from multiplying by various mechanisms of action [39,40,41,42].

## 3. Flavonoids Target EBV and Its Correlated Cancers

EBV is a widespread human herpesvirus that persists latently in infected cells lifelong, and is commonly transmitted via infected saliva [43,44]. The lytic reactivation of EBV has clinically been recognized to be linked to several types of cancer, such as nasopharyngeal carcinoma (NPC), T-cell lymphoma, Hodgkin lymphoma, Burkitt’s lymphoma, and gastric cancer [45,46,47,48]. In addition, EBV is the causative agent of infectious mononucleosis (kissing disease) [49]. EBV encodes various genes, proteins, and antigens that are typically expressed during the lytic cycle, latent phase, reactivation, and formation of the linked tumors. Subsequently, the expressed genes, proteins, and antigens boost cell growth, transformation, and metabolic reprogramming, leading to cancer invasion [13,50,51,52]. Therefore, blocking the infection of EBV by targeting various steps of its life cycle, including the lytic cycle, the expression of latent and lytic products (genes, proteins, and antigens), and other implicated signaling pathways could be an effective approach for managing EBV-associated cancers [3,53,54].

### 3.1. Flavonoids with Anti-EBV Properties

**Figure 2:**
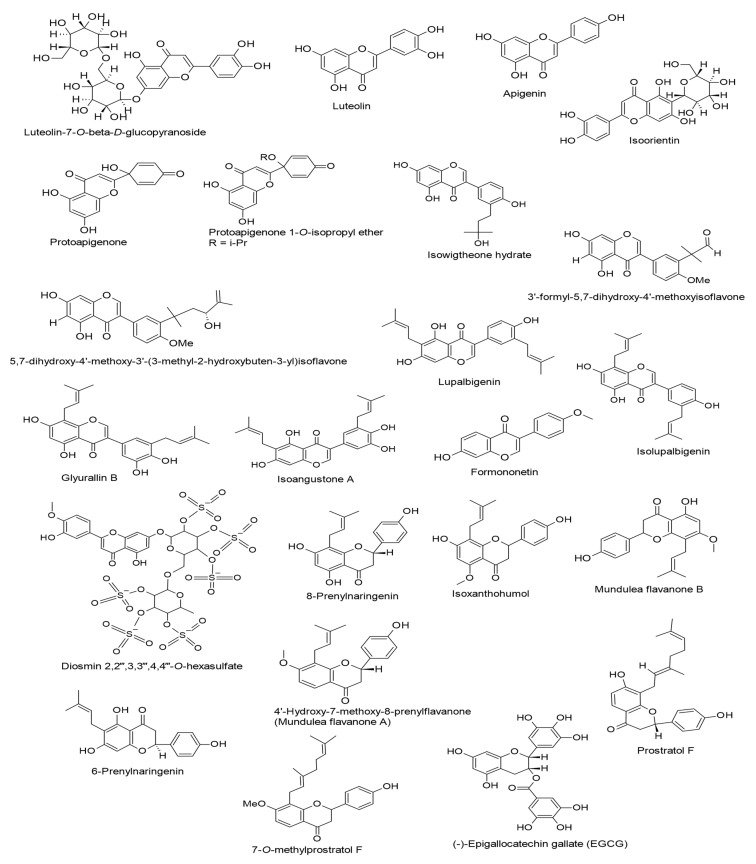

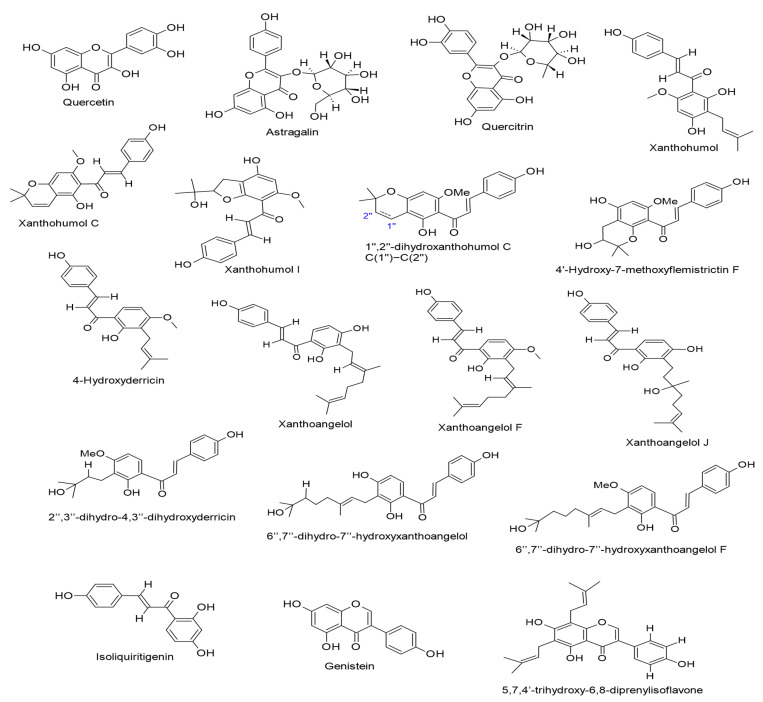
Chemical structures of flavonoids (flavones, protoflavones, isoflavones, flavanones, catechins, flavonols, and chalcones) with inhibitory activities against Epstein–Barr virus (EBV).

In Table 1, we outline various flavonoid-type compounds reported to induce inhibitory activities against EBV evaluated in vitro and in silico. Their chemical structures are shown in Figure 2. As displayed in the table and according to [55,56,57,58,59,60,61,62,63,64,65,66,67,68,69,70,71,72], the presented molecules were detected to affect diverse stages of the EBV life cycle, such as viral entry, lytic replication, DNA load, virion production, and latency by various mechanisms, via inhibiting the expressions of glycoprotein H (gH), replication and transcription activator (Rta), an immediate-early gene (Zta), transcription factor specificity protein 1 (SP1), latent membrane protein 1 (LMP1), EBV early antigen (EBV-EA), EBV nuclear antigen 1 (EBNA1), early antigen D (EA-D), and viral capsid antigen (VCA), and by downregulating the signaling pathways of mitogen-activated protein kinase–kinase/extracellular signal-regulated kinase 1/2 (MEK/ERK1/2), phosphatidylinositol 3-kinase/protein kinase B (PI3K/AKT), mitogen-activated protein kinases/wild-type p53 (MAPKs/wt-p53), and c-Jun N-terminal kinases/c-Jun (JNKs/c-Jun).

Importantly, quercetin was found to inhibit an EBV infection with the ability to adversely impact this effect by promoting lytic reactivation [72]. Similarly, Huh et al. [73] also pointed out this adverse action, where quercetin was noticed to upregulate the expression of EBV lytic gene promoter BHLF1, indicating the potential negative impact on blocking EBV infection. Therefore, further investigations are required to evaluate the influencing factors that lead to such an effect.

Ito et al. [62] reported that the prenyl group on the A-ring, the prenyl group at 3′, and a hydroxyl group at 4′ placed on the isoflavone skeletons of lupalbigenin, isolupalbigenin, glyurallin B, and isoangustone A are responsible for the induced inhibitory actions against EBV-EA; according to the structure–activity relationship evaluation. Moreover, the same research group showed in a previous study the importance of the presence of a prenylated isoflavone analog as a contributing factor accountable for the anti-EBV-EA activity of some examined isoflavones isolated from *Millettia atropurpurea*, such as 5,7,4′-trihydroxy-6,8-diprenylisoflavone (100% inhibition at 1 × 10^3^ mol ratio/12-*O*-tetradecanoylphorbol-13-acetate (TPA)) [74]. The chemical synthesis of natural products plays a fundamental role in drug discovery [75]. In this context, an earlier study presented a synthetic analog PJ-2–genistein conjugate consisting of genistein (an isoflavone) and 3*β*-methoxyserrat-14-en-21*β*-ol (PJ-2) with a promising anti-EBV effect by targeting EBV-EA. The synthesized compound displayed inhibitory action against EBV-EA activation with a 50% inhibitory concentration (IC_50_) value of 455 mol ratio/32 pmol TPA, compared with the drug control oleanolic acid (IC_50_ = 449 mol ratio/32 pmol TPA) [76].

### 3.2. Therapeutic Effects of Flavonoids against EBV-Associated Cancers

#### 3.2.1. Flavones and Prenylated Flavonols

Luteolin is a bioactive flavone distributed in various medicinal plants, fruits, and vegetables with diverse biological properties, including anticancer and antiviral activities [77,78,79]. Several experimental studies indicated the protective potential of luteolin against different types of cancer correlated with EBV infection with diverse mechanisms. For instance, Wu et al. [80] reported the results of a combined in vitro and in vivo study to reveal the effect of luteolin on NPC performed on EBV-positive NPC cells. Luteolin (0–50 µM) was discovered to inhibit EBV reactivation by repressing Rta and Zta gene expressions and lessening genomic instability caused by recurrent EBV reactivation in NPC cells. It also suppressed cell proliferation, migration, invasion, and spheroid formation (at concentrations ranging from 10 to 50 µM). Furthermore, tumor growth in a mouse model induced by EBV reactivation was effectively repressed by luteolin treatment (40 mg/kg administered intraperitoneally every 3 or 4 days for 4 weeks).

EBV-LMP1 is known to promote cell proliferation and progression of NPC cells [81]. According to the findings obtained by Lo and colleagues [82], a new function of LMP1 (expressed in EBV-positive NPC cells) in enhancing lipogenesis was discovered. LMP1 was observed to elevate the expression, maturation, and activation of sterol regulatory element-binding protein 1 (SREBP1), a main regulator of lipogenesis, and its downstream target fatty acid synthase (FASN). Remarkably, luteolin (20 µM) has notably hindered the lipogenesis and proliferation of NPC cells by suppressing LMP1, SREBP1, and FASN expressions. Moreover, luteolin has effectively inhibited NPC growth and induced apoptosis in mice at a dose of 20 mg/kg administered every 2–3 days for 3 weeks.

Another study demonstrated the promising anticancer effect of luteolin against NPC, by preventing the infection of EBV by targeting lytic replication. The underlying mechanism was explored by impeding Rta and Zta expressions and downregulating SP1 activity [56].

The molecular mechanisms of wogonin, isolated from *Scutellaria baicalensis* Georgi., against EBV-infected lymphoma were explored using in vitro and animal models. This compound (50 µM) expressed cell apoptosis by downregulating the expression of nuclear factor-κB (NF-κB), by targeting the LMP1/miR-155/NF-κB/PU.1 pathway. In addition, wogonin (8 mg/kg/2 days for two weeks) repressed tumor growth in mice xenograft models infested with EBV-infected lymphoma, via downregulating ki67 and p65 expressions and upregulating PU.1 expression [83].

Treatment of EBV-positive NPC cells with baicalein, a bioactive constituent of *Scutellaria baicalensis*, at concentrations of 0, 15, 30, and 60 µM, significantly diminished the expressions of Sp1, EBNA1, and EBNA1 Q-promoter. Moreover, in a mouse xenograft model of EBV-positive NPC, baicalein has notably decreased tumor progression at a low dose of 15 mg/kg/day or a high dose of 30 mg/kg/day for 21 days [84]. By targeting EBV-positive B-cell tumors, additional anticancer mechanisms of baicalein (100 µM) were determined in vitro. Baicalein-mediated signal-regulating kinase 1/c-Jun N-terminal kinase (ASK1/JNK) activation was found to regulate the mitochondria-dependent apoptosis pathway by upregulating transcriptionally active p63 (TAp63) expression and downregulating NF-κB and proteins CD74/CD44 expressions [85]. 

**Figure 3:**
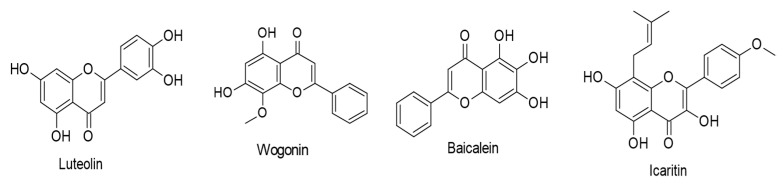
Chemical structures of flavones and prenylated flavonols with therapeutic actions against EBV-associated cancers.

Wu and coworkers [86] studied in vitro the therapeutic potential and underlying molecular mechanisms of icaritin, a major prenylated flavonol of the *Epimedium* genus, against EBV-positive extranodal NK/T-cell lymphoma (ENKL), an aggressive hematological tumor. The acquired results showed that icaritin (16–50 µM) impedes ENKL cell proliferation and generates apoptosis and cell cycle arrest at the G_2_/M phase. It was also found to upregulate Bax (pro-apoptotic protein), downregulate Bcl-2 (anti-apoptotic protein) and pBad (protein Bad), and activate caspase-3 and caspase-9. The mechanism responsible for the induced anti-proliferative and pro-apoptotic activities was ascertained to be mediated by suppressing signal transducer and activator of transcription 3 (STAT3) and AKT pathways through LMP1 downregulation. Significantly, combined treatment of icaritin (50 µM) with the antiviral drug ganciclovir (25 µg/mL) induced potent ENKL cell apoptosis. The chemical structures of the reviewed flavones and prenylated flavonols are shown in Figure 3.

#### 3.2.2. Isoflavones

Bioassay-guided fractionation of the extract of *Ficus hispida* L.f. led to the isolation of three isoflavone-type molecules, isowigtheone hydrate, 3′-formyl-5,7-dihydroxy-4′-methoxyisoflavone, and 5,7-dihydroxy-4′-methoxy-3′-(3-methyl-2-hydroxybuten-3-yl)isoflavone with anti-EBV-EA activities determined at IC_50_ values of 271, 358, and 285 mol ratio/32 pmol TPA, respectively. Further, an in vitro anticancer screening of the isolated compounds showed potent cytotoxic effects on human cancer cells, such as breast carcinoma and cervical adenocarcinoma, which associate with EBV infection (IC_50_ 10.8–60.2 µM). Among the test compounds, isowigtheone hydrate induced reactive oxygen species (ROS) production, guiding to activating caspases-3, -8, and -9 apoptotic processes [61].

**Figure 4:**
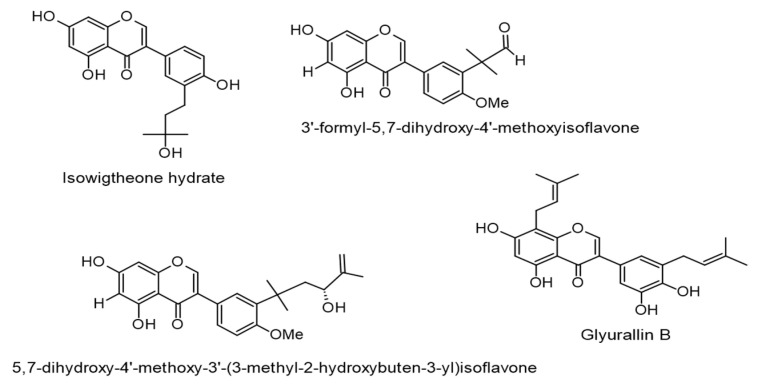
Chemical structures of isoflavones with therapeutic effects against EBV-associated cancers.

Glyurallin B, an isoflavone isolated from *Derris scandens* (Roxb.) Benth., demonstrated inhibitory actions against EBV and its associated squamous cell carcinoma, also known as epidermoid carcinoma. Glyurallin B efficiently inhibited an EBV-EA activation with an IC_50_ value of 278 mol ratio/32 pmol TPA. It also exposed in vitro cytotoxicity and antiproliferative activities against squamous cell carcinoma at a concentration of 5 µM. The underlying mechanism was observed to relate to disturbing the mitochondrial pathway [62]. The chemical structures of the reviewed isoflavones are displayed in Figure 4.

#### 3.2.3. Flavanones

Akihisa et al. [66] have performed a chemical modification on prenylated chalcones (4-hydroxyderricin, xanthoangelol, and xanthoangelol F; extracted from *Angelica keiskei*) employing microbial transformation by the fungus *Aspergillus saitoi* to produce three flavanone-type compounds, 4′-Hydroxy-7-methoxy-8-prenylflavanone (Mundulea flavanone A), prostratol F, and 7-*O*-methylprostratol F, with antitumor-promoting activities against the induction of EBV-EA. The transformed compounds exhibited anti-EBV-EA activity with IC_50_ values of 230, 270, and 348 mol ratio/32 pmol TPA, respectively. Additionally, treatment of human cancer cells (leukemia and melanoma) with the test flavanones induced considerable cytotoxic effects, with IC_50_ values ranging from 7.2 to 17.0 µM.

**Figure 5:**
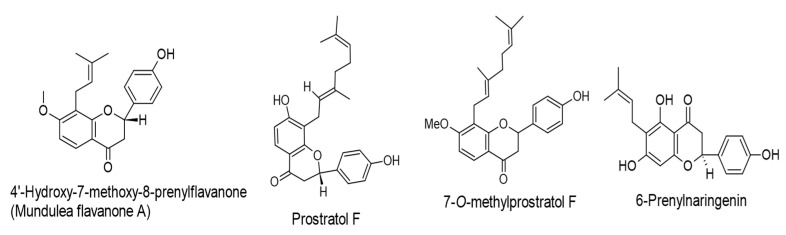
Chemical structures of flavanones with therapeutic properties against EBV-associated cancers.

6-Prenylnaringenin (85 nmol), a flavanone obtained from *Humulus lupulus* L., displayed notable inhibitory properties against skin-tumor promotion in an in vivo two-stage mouse-skin carcinogenesis. Moreover, this compound in vitro induced anti-EBV-EA properties with an IC_50_ value of 263 mol ratio/32 pmol TPA [65]. The chemical structures of the reviewed flavanones are presented in Figure 5.

#### 3.2.4. Flavonols

Quercetin is a biologically active flavonol identified in various vegetables and fruits, including lovage, cilantro, dill, onions, apples, and berries. It possesses multiple bioactivities against a wide range of diseases, including cancer and viral infections [87,88,89]. The direct anticancer activity of quercetin on EBV-associated cancers has been revealed in numerous laboratory and animal experiments with various mechanisms. For example, at an effective dose of 30 mg/kg/day for 2 weeks, quercetin displayed a potent anticancer effect in mice xenograft models infected with EBV-human gastric carcinoma. The molecular mechanism responsible for the induced anticancer action was explored by interfering with the expressions of viral EBNA1 and LMP2. Moreover, quercetin has significantly generated p53-dependent apoptosis in EBV-human gastric carcinoma, which was found to be linked with overexpression of the cleaved forms of caspase-3, -9, and Parp [90]. An additional in vitro experiment showed the capacity of quercetin (62 µM) to induce cytotoxic effect, apoptosis, and cell cycle arrest against EBV-associated gastric carcinoma cells. It also prevented an EBV infection by interacting with two steps of the viral life cycle, such as entry and latency, and inhibiting the expression of EBNA1 at a concentration of 62 µM [72].

In further in vivo study, the antitumor action of quercetin against EBV-associated gastric carcinoma performed on a mouse xenograft model was enhanced via a co-treatment with *Ganoderma lucidum* extract, administered at effective doses of 10 mg/kg/23 days for quercetin and 10 mg/kg/23 days for *Ganoderma lucidum* extract [73].

Granato et al. [91] detailed in vitro the cytotoxic effect of quercetin (100 µM) against EBV-positive Burkitt’s lymphoma cells by decreasing cellular myelocytomatosis oncogene (c-Myc) expression and downregulating the PI3K/AKT/mTOR signaling pathway, leading to causing apoptosis.

The chemopreventive activity of quercetin (100 µM) against NPC was reported in vitro via inhibiting cell proliferation and reducing the expression of FASN in EBV-positive NPC cells [92]. In any case, further protective action of quercetin (85 nmol; used as standard drug) against papilloma formation in two-stage mouse skin carcinogenesis was unveiled [93].

During EBV lytic reactivation, several oncogenic pathways are activated to promote viral carcinogenesis. Consequently, Granato et al. [94] conducted an in vitro investigation using quercetin to explore the possibility of inhibiting these pathways. The results indicated that quercetin (10 µM) could suppress the activation of STAT3 pathway generated by EBV infection and diminish the production of interleukin-6 (IL-6) and ROS. Interestingly, quercetin was observed to stimulate autophagy and neutralize the accumulation of sequestosome1/p62 (SQSTM1/p62), leading to stopping B cell immortalization. The study authors concluded that quercetin is a promising natural anticancer agent for treating EBV-associated malignancies and counteracting EBV-driven lymphomagenesis.

**Figure 6:**
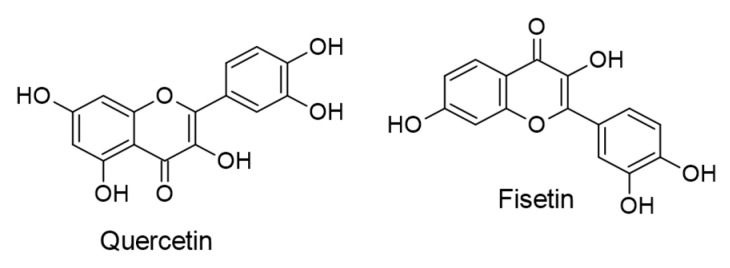
Chemical structures of flavonols with therapeutic actions against EBV-associated cancers.

Fisetin, a flavonol molecule, has been isolated from numerous fruits, vegetables, and medicinal trees with proven dietary and pharmacological properties against several health conditions [95,96]. Its efficacy against cancers developed by EBV infections has been reported in some investigations. At various concentrations in micromolar ranges, fisetin in vitro prevented the metastasis of NPC generated by EBV infection by multiple mechanisms through suppressing the migration and invasion of LMP1-expressing NPC cells. The mechanisms were mediated via repressing molecular changes linked with the epithelial-mesenchymal transition (EMT) promoted by LMP1 and upregulating the epithelial marker and E-cadherin protein. Moreover, the levels of mesenchymal marker, vimentin protein, and twist protein (an EMT regulator) were successfully downregulated by fisetin [97]. Similarly, Li et al. [98] elucidated in vitro other mechanisms by which fisetin regulates cancer growth examined in EBV-infected NPC cells. According to their finding, fisetin (6.25–100 µM) was noted to interfere with targets of the NF-κB signal transduction pathway (p65, IκBα, and CyclinD1) activated by EBV-LMP1. The chemical structures of the reviewed flavonols are shown in Figure 6.

#### 3.2.5. Dihydroflavonols

**Figure 7:**
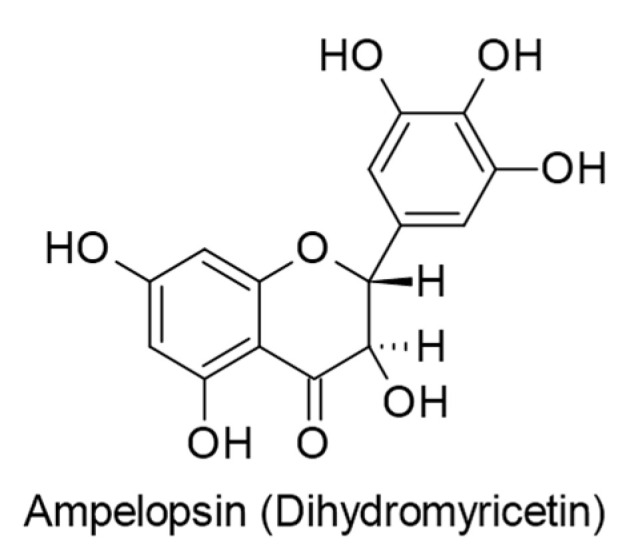
Chemical structure of dihydroflavonol ampelopsin with therapeutic effect against EBV-positive human Burkitt’s lymphoma cells.

Yun and colleagues [99] explored the anticancer mechanism of action of ampelopsin (also known as dihydromyricetin; Figure 7), an active constituent of *Ampelopsis grossedentata*, against EBV-positive human Burkitt’s lymphoma cells. The results of in vitro assays indicated that ampelopsin (0–50 µM) caused the apoptosis of EBV-infected cells via upregulating tumor necrosis factor-related apoptosis-inducing ligand/death receptor 5 (TRAIL/DR5) and activating the p38 signaling pathway.

#### 3.2.6. Chalcones

Two prenyl-chain-hydrated chalcones, 2″,3″-dihydro-4,3″-dihydroxyderricin and 6″,7″-dihydro-7″-hydroxyxanthoangelol F were chemically modified from prenylated chalcones (4-hydroxyderricin, xanthoangelol, and xanthoangelol F; isolated from *Angelica keiskei*) using biotransformation by the fungus *Aspergillus saitoi*. The transformed molecules possessed anti-tumor-promoting actions against an EBV-EA activation, with IC_50_ values of 210 and 215 mol ratio/32 pmol TPA, respectively. 2″,3″-dihydro-4,3″-dihydroxyderricin demonstrated potent cytotoxicity against human leukemia cells by forming apoptosis with an IC_50_ value of 2.9 µM. Moreover, inhibitory action against skin tumor promotion in an in vivo two-stage mouse skin carcinogenesis was effectively induced by 6″,7″-dihydro-7″-hydroxyxanthoangelol F (85 nmol) [66].

**Figure 8:**
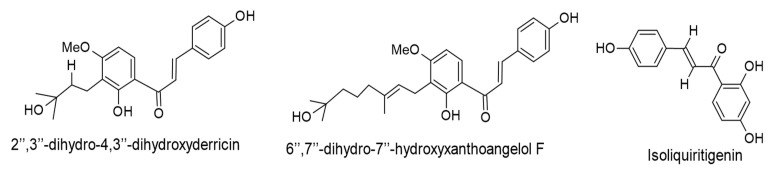
Chemical structures of chalcones with therapeutic effects against EBV-correlated cancers.

Isoliquiritigenin is a chalcone-type molecule known to cause cell cycle arrest and apoptosis of various cancer cells. This compound is commonly scattered in various species of the genus *Glycyrrhiza*, including *Glycyrrhiza uralensis*, *Glycyrrhiza radix*, and *Glycyrrhiza glabra* [100,101]. In an in vitro assay, isoliquiritigenin (45 µM) demonstrated antitumor properties against EBV-associated gastric carcinoma cells by causing cytotoxicity, apoptosis, and cell cycle arrest [72]. Another in vitro investigation showed the capacity of isoliquiritigenin (50 µM) to induce cytotoxic and antiproliferative effects on EBV-transformed lymphoblastoid cell lines through molecular mechanisms that involve microRNAs by targeting the p53 signaling pathway and cell cycle [102]. Figure 8 displays the chemical structures of the reviewed chalcones.

#### 3.2.7. Other Flavonoid-Type Compounds

**Figure 9:**
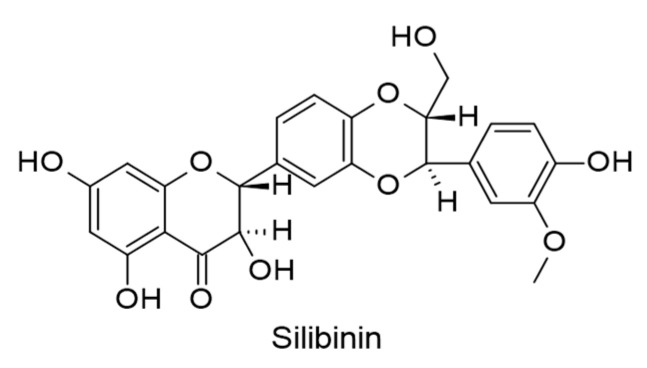
Chemical structure of silibinin with therapeutic action against EBV-positive nasopharyngeal carcinoma (NPC) cells.

Silibinin (Figure 9), a flavonolignan, has been extracted from *Silybum marianum* (L.) Gaertn. with potential therapeutic effect on clinically isolated EBV-positive NPC cells. Silibinin (0–200 µM) remarkably downregulated programmed death-ligand 1 (PD-L1) expression by inhibiting hypoxia-inducible factor-1α/lactate dehydrogenase-A (HIF-1α/LDH-A) mediated cell metabolism in EBV-positive NPC [103].

## 4. Flavonoids Target KSHV and Its Correlated Cancers

KSHV is a pathogenic gamma-herpesvirus that was first identified in Kaposi sarcoma (KS), a cancer that develops from the cells that line lymph or blood vessels. This virus is also associated with other cancerous tumors, such as primary effusion lymphoma (PEL) and various B-cell cancers. The virus is transmitted by sex, blood, and infected saliva [2,104,105,106]. Numerous genes and proteins are encoded by KSHV, which play a critical role in the viral life cycle and contribute to cell transformation, induction, and progression of the associated malignancies [14,107,108,109]. Therefore, new strategies for managing KSHV-related malignancies were recently proposed by targeting the KSHV life cycle (mainly the lytic reactivation) and the expressions of latent and lytic genes/proteins along with other correlated signaling pathways [3,110,111].

### 4.1. Flavonoids with Anti-KSHV Properties

**Figure 10:**
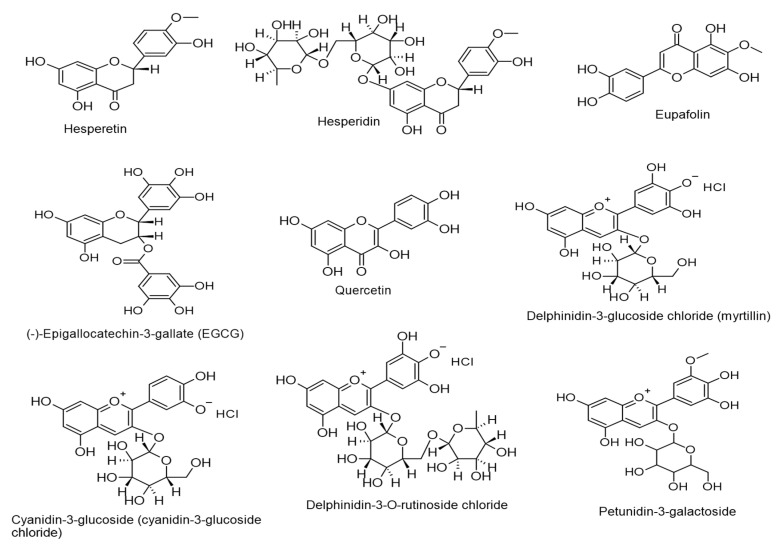
Chemical structures of flavonoids (flavanones, flavones, catechins, flavonols, and anthocyanins) with inhibitory actions against Kaposi sarcoma-associated herpesvirus (KSHV).

Over the past ten years, the direct inhibitory effect of flavonoids on the KSHV life cycle has been reported in a decent amount of studies. In Table 2, we document these findings that feature the anti-KHSV efficacy of flavonoids, and their chemical structures are displayed in Figure 10. According to the results obtained by [112,113,114,115,116], flavonoids were found to target the KSHV life cycle by various mechanisms via impeding the lytic DNA replication, virus progeny production, and the expressions of hypoxia-inducible factor 1α (HIF1α), immediate-early protein ORF45, late protein K8.1, and Rta. Moreover, targeting ROS was described as a mechanism that suppresses the KSHV lytic cycle.

### 4.2. Therapeutic Effects of Flavonoids against KSHV-Associated Cancers

#### 4.2.1. Flavones

Zhu et al. [117] studied, in vitro and in vivo, the potential anticancer properties of oroxylin A, an active ingredient of *Oroxylum indicum*, against KSHV-associated cancers, including KS and other aggressive angiomas. They found that oroxylin A (50 µM) inhibited lymphatic reprogramming of the endothelial cells generated by KSHV oncogene vIL-6, leading to cell invasion blocking, apoptosis, and neovascularization inhibition via modulating PPARγ/Prox1 axis.

**Figure 11:**
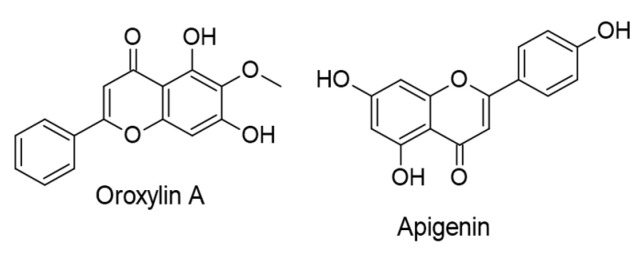
Chemical structures of flavones with therapeutic effects against KSHV-associated malignancies.

By utilizing KSHV-positive PEL cells assayed in vitro, apigenin (12.5 and 25 µM) exposed anticancer activity by causing PEL cell death and autophagy, as well as by reducing intracellular ROS production. The mechanisms were determined by activating p53, which promotes catalase activity (a ROS scavenger enzyme) and impeding STAT3, a critical pro-survival pathway in PEL [118]. The chemical structures of the reviewed flavones are presented in Figure 11.

#### 4.2.2. Flavonols

**Figure 12:**
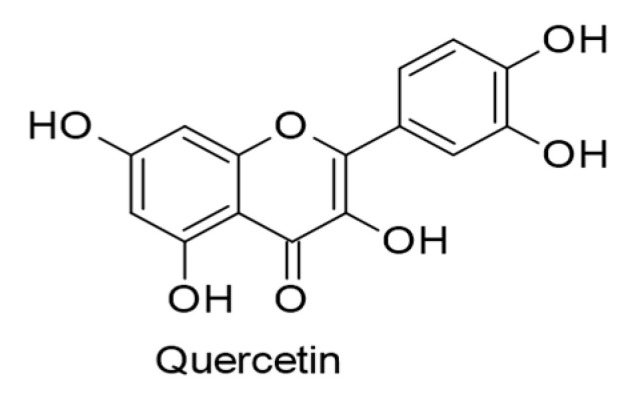
Chemical structure of quercetin with therapeutic effect against KSHV-positive primary effusion lymphoma (PEL) cells.

At a concentration of 50 µM, quercetin (Figure 12) in vitro was observed to interfere with multiple cancer-related pathways, inducing apoptosis and autophagy in KSHV-positive PEL cells. It repressed PI3K/AKT/mTOR and STAT3 signaling pathways and downregulated the expression of the pro-survival cellular proteins such as c-FLIP, cyclin D, and cMyc. Quercetin was also found to reduce the expression of lytic KSHV proteins implicated in PEL tumorigenesis, such as K-bZIP, and upregulate HLA-DR and calreticulin expressions. Importantly, quercetin caused pro-survival autophagy in KSHV-positive PEL cells and improved the cytotoxic action of bortezomib, a proteasome inhibitor, against these cells [119].

Another anticancer mechanism of quercetin was explored against KSHV-positive PEL cells using in vitro experiments. Quercetin (15 µM) effectively reduced ROS production by downregulating p62/Sequestosome1 (p62/SQSTM1) protein expression, leading to cell apoptosis and inhibition of KSHV lytic replication [115]. Further in vitro research disclosed the ability of quercetin (10 µM) to decrease the level of ROS production, specifically H_2_O_2_, in KSHV-positive PEL cells, leading to inhibiting the KSHV lytic cycle and boosting cell death [120].

#### 4.2.3. Catechins

**Figure 13:**
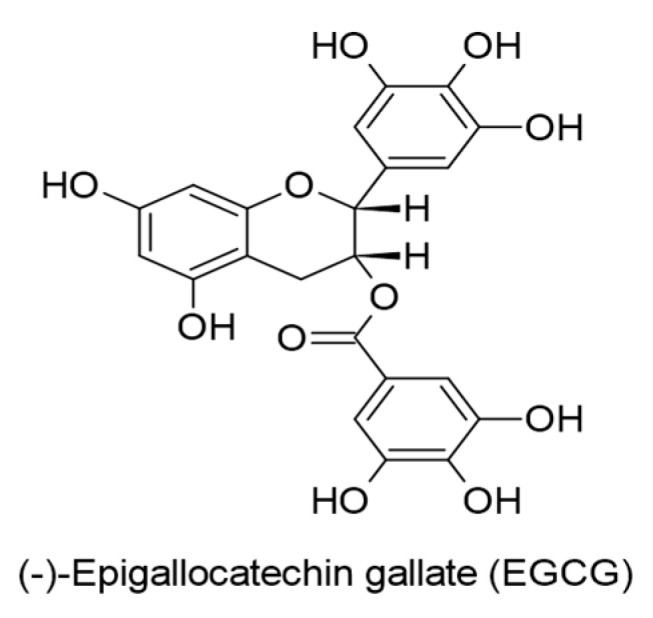
Chemical structures of (-)-Epigallocatechin gallate with therapeutic action against KSHV-positive primary effusion lymphoma (PEL) cells.

EGCG (Figure 13), a principal compound of *Camellia sinensis*, has proven cancer chemopreventive properties against KSHV-infected PEL cells, and its molecular mechanisms were detailed using in vitro experiments. EGCG caused cell cycle arrest and apoptosis in KSHV-infected PEL cells in a dose-dependent manner (20 µg/mL) by a mechanism that regulates ROS generation [114]. Additional mechanisms were reported by Yeh et al. [121], where EGCG (20 µg/mL) induced cell death in KSHV-infected PEL cells by upregulating glucose transporter GLUT3 expression and decreasing the expressions of pyruvate dehydrogenase E1-alpha (PDHA1), the main regulator of pyruvate dehydrogenase (PDH), and glutamate dehydrogenase 1 (GDH1). In addition, EGCG lessened the levels of oncometabolite D-2-hydroxyglutarate (D2HG).

## 5. Conclusions and Future Directions

The incidence of infectious diseases has become one of the most significant global health threats of the 21st century; this has been observed during the latest pandemic of coronavirus disease 2019 (COVID-19). Moreover, studies of viral infections that lead to cancer have become one of the most important goals in modern medicine, where cancer is a leading cause of death. For years, many hurdles, including the problem of drug resistance, have restricted achieving efficient treatments for viral diseases and cancer. Therefore, new medications are necessary. Flavonoids are a rich source of compounds with structural and chemical diversity for drug discovery. In this paper, we provided a critical assessment of the protective capability of flavonoids and how they might manage human gamma-herpesvirus infections and their associated tumors by diverse molecular mechanisms. The reviewed flavonoids were examined by different experimental methodologies performed in various in vitro, in vivo, and in silico models. In addition, this review emphasized several strategies to improve the efficacy of flavonoids against EBV and its linked cancers, such as chemical synthesis, microbial transformation, and combinatory treatment with standard antiviral drugs such as ganciclovir. Moreover, quercetin in combination with bortezomib, a proteasome inhibitor, boosted the cytotoxicity against KSHV-positive PEL cells and, hence, improved treatment efficacy. The molecular mechanisms of luteolin, baicalein, wogonin, 6-Prenylnaringenin, quercetin, and 6″,7″-dihydro-7″-hydroxyxanthoangelol F against EBV-associated tumors have been validated by animal experiments. In addition, the effectiveness of oroxylin A against KSHV-related malignancies, including KS, was confirmed in an animal study. Accordingly, these compounds merit special consideration to be engaged in more animal investigations and subsequently in clinical studies.

Concerning future directions, investigations on nano-drug delivery combined with pharmacokinetic and pharmacodynamic evaluations should be developed to improve the bioavailability and the activities of the reviewed flavonoids against EBV, KSHV, and their connected tumors. When it comes to clinical application, the involvement of the reviewed flavonoids in clinical studies faces various challenges. Aside from other obstacles related to financial support, the challenging regulatory requirements, and among others, the safety profile of the test compound, its bioavailability, and finding a sustainable and economically viable supply of sufficient quantities of the test compound are limiting factors. So far, no clinical trials have been conducted on flavonoids against human gamma-herpesviruses; however, dietary flavonoids such as quercetin, EGCG, and luteolin have proven sufficient effectiveness in preliminary clinical investigations against various types of cancer, including malignancies that might be generated by gamma-herpesvirus infections. In general, the participation of flavonoids in clinical studies is still challenging, and more efforts should be made to achieve this goal.

## Figures and Tables

**Table 1:** Flavonoids with anti-EBV properties and their molecular mechanisms assayed in vitro and in silico.

Compound, Concentration, and Source	Chemical Class	Molecular Mechanisms (Inhibition/Downregulation)	Reference
Luteolin-7-*O*-*β*-*D*-glucopyranoside. (20 µg/mL). *Lindernia Crustacea*.	Flavones	Lytic replication. Rta expression.	[55]
Luteolin. (10, 20, and 50 µM). Various medicinal herbs, fruits, and vegetables.	Flavones	Lytic replication. Rta and Zta expressions. Sp1-luc activity.	[56]
Apigenin. (50 µM). Distributed in various fruits and vegetables.	Flavones	Lytic replication. Virion production. Rta and Zta expressions.	[57]
Isoorientin (An IC_50_ value of 393 mol ratio/32 pmol TPA). *Passiflora edulis*.	Flavones	Lytic cycle. EBV-EA.	[58]
Protoapigenone. (0.31 mM). *Thelypteris torresiana*.	Protoflavones	Lytic cycle. Rta, Zta, EA-D, and VCA expressions.	[59]
Protoapigenone. (IC_50_ = 0.127 µM and 0.50 µM). *Thelypteris torresiana*	Protoflavones	Lytic replication. Rta expression.	[60]
Protoapigenone 1′-*O*-isopropyl ether. (IC_50_ = 0.467 µM and 0.25 µM). Synthatically derived from apigenin.	Protoflavones	Lytic replication. Rta expression.	[60]
Isowigtheone hydrate, 3′-formyl-5,7-dihydroxy-4′-methoxyisoflavone, and 5,7-dihydroxy-4′-methoxy-3′-(3-methyl-2-hydroxybuten-3-yl)isoflavone. (IC_50_ values of 271, 358, and 285 mol ratio/32 pmol TPA, respectively). *Ficus hispida* L.f.	Isoflavones	Lytic cycle. EBV-EA.	[61]
Lupalbigenin, isolupalbigenin, glyurallin B, and isoangustone A. (IC_50_ values of 290, 285, 278, and 282 mol ratio/32 pmol TPA, respectively). *Derris Scandens* (Roxb.) Benth.	Isoflavones	Lytic cycle. EBV-EA.	[62]
Formononetin. Binding affinity (−6.6 kcal/mol). PubChem CID: 5280378.	Isoflavones	EBV life cycle (entry). EBV gH (in silico).	[63]
Diosmin 2″,2‴,3″,3‴,4″,4‴-*O*-hexasulfate. (20 µM). Binding affinity with Zta (−8.7 kcal/mol). Synthesized.	Flavones (Sulfated)	Lytic replication. EBV DNA load. LMP1. Zta (in silico).	[64]
8-Prenylnaringenin, isoxanthohumol, mundulea flavanone B, and 6-Prenylnaringenin. (IC_50_ values of 263, 293, 281, and 263 mol ratio/32 pmol TPA, respectively). *Humulus lupulus* L.	Flavanones	Lytic cycle. EBV-EA.	[65]
4′-Hydroxy-7-methoxy-8-prenylflavanone (Mundulea flavanone A), prostratol F, and 7-*O*-methylprostratol F. (IC_50_ values of 230, 270, and 348 mol ratio/32 pmol TPA, respectively). Biotransformed products of prenylated chalcones by *Aspergillus saitoi*.	Flavanones	Lytic cycle. EBV-EA.	[66]
		Lytic replication. MEK/ERK1/2. PI3K/AKT.	[67]
		Lytic replication. LMP1.	[68]
(-)-Epigallocatechin gallate (EGCG). (0.5-50 µM). Binding affinity with EBNA1 (−6.8 kcal/mol). Binding affinity with gH (−7.8 kcal/mol). *Camellia sinensis*.	Flavanols (Catechins)	Lytic replication. LMP1. MAPKs/wt-p53. JNKs/c-Jun.	[69]
		Lytic replication. EBNA1. oriP-DNA.	[70]
		Lytic replication. EBNA1 (in silico).	[71]
		EBV life cycle (entry). EBV gH (in silico).	[63]
Quercetin. (62 µM). *Glycyrrhiza uralensis*.	Flavonols	EBV life cycle (entry and latency). EBNA1.	[72]
Astragalin and quercitrin. (IC_50_ values of 543 and 532 mol ratio/32 pmol TPA, respectively). *Humulus lupulus* L.	Flavonols (Flavonol glycosides)	Lytic cycle. EBV-EA.	[65]
Xanthohumol, Xanthohumol C, 1″,2″-dihydroxanthohumol C, Xanthohumol I, and 4′-Hydroxy-7-methoxyflemistrictin F. (IC_50_ values of 485, 520, 526, 470, and 501 mol ratio/32 pmol TPA, respectively). *Humulus lupulus* L.	Chalcones	Lytic cycle. EBV-EA.	[65]
4-Hydroxyderricin, xanthoangelol, xanthoangelol F, 2″,3″-dihydro-4,3″-dihydroxyderricin, 6″,7″-dihydro-7″-hydroxyxanthoangelol, xanthoangelol J, and 6″,7″-dihydro-7″-hydroxyxanthoangelol F. (IC_50_ values of 213, 269, 262, 210, 211, 219, and 215 mol ratio/32 pmol TPA, respectively). The first three compounds were isolated from *Angelica keiskei*, while the rest are biotransformed products of prenylated chalcones by *Aspergillus saitoi*.	Chalcones	Lytic cycle. EBV-EA.	[66]
Isoliquiritigenin. (45 µM). *Glycyrrhiza uralensis*.	Chalcones	EBV entry.	[72]

Abbreviations: BHLF1, lytic gene promoter; DNA, deoxyribonucleic acid; EA-D, early antigen D; EBNA1, EBV nuclear antigen 1; EBV, Epstein–Barr virus; EBV-EA, EBV early antigen; ERK1/2, extracellular signal-regulated kinase 1/2; gH, glycoprotein H; IC_50_, 50% inhibitory concentration; JNKs/c-Jun, c-Jun N-terminal kinases/c-Jun; LMP1, latent membrane protein 1; MAPKs/wt-p53, mitogen-activated protein kinases/wild-type p53; MEK, mitogen-activated protein kinase–kinase; oriP-DNA, an origin of DNA replication; PI3K/AKT, phosphatidylinositol 3-kinase/protein kinase B; Rta, replication and transcription activator; SP1, transcription factor specificity protein 1; TPA, 12-*O*-tetradecanoylphorbol-13-acetate; VCA, viral capsid antigen; Zta, an immediate-early gene. Important note: The displayed results were assayed in vitro, and the findings that were examined in silico are highlighted in parentheses in the table.

**Table 2:** Flavonoids with anti-KSHV properties and their molecular mechanisms evaluated in vitro.

Compound, Concentration, and Source	Chemical Class	Molecular Mechanisms (Inhibition)	Reference
Hesperetin. (0.5–50 µM). Distributed in the genus *Citrus*.	Flavanones	Lytic DNA replication. Virus progeny production. HIF1α expression.	[112]
Hesperidin. (EC_50_ = 0.2399 µM and 18 µM). *Thymus capitatus*.	Flavanones	Lytic DNA replication. ORF45 and K8.1 expressions.	[113]
Eupafolin. (EC_50_ = 1.396 µM and 105 µM). *Thymus capitatus*.	Flavones	Lytic DNA replication. ORF45 and K8.1 expressions.	[113]
(-)-Epigallocatechin gallate (EGCG). (50 µg/mL). *Camellia sinensis*.	Flavanols (Catechins)	Lytic DNA replication. Virus progeny production. Rta expression.	[114]
Quercetin. (15 µM). Fruits and vegetables.	Flavonols	Lytic DNA replication. ROS.	[115]
Delphinidin-3-glucoside chloride (myrtillin), cyanidin-3-glucoside, delphinidin-3-*O*-rutinoside chloride, and petunidin-3-galactoside. (75 and 150 µg/mL). *Ribes nigrum* L. *Vaccinium myrtillus* L.	Anthocyanins	Lytic DNA replication.	[116]

Abbreviations: EC_50_, 50% effective concentration; DNA, deoxyribonucleic acid; HIF1α, hypoxia-inducible factor 1α; K8.1, late protein; ORF45, immediate-early protein; ROS, reactive oxygen species; Rta, replication and transcription activator.

## Data Availability

Not applicable.
